# Microbiota-gut-brain axis and the central nervous system

**DOI:** 10.18632/oncotarget.17754

**Published:** 2017-05-10

**Authors:** Xiqun Zhu, Yong Han, Jing Du, Renzhong Liu, Ketao Jin, Wei Yi

**Affiliations:** ^1^ Department of Neurosurgery, Renmin Hospital of Wuhan University, Wuhan, Hubei, P.R. China; ^2^ Clinical Research Institute, Zhejiang Provincial People's Hospital, Hangzhou, Zhejiang, P.R. China; ^3^ Department of Gastroenterology, Zhejiang Provincial People's Hospital, Hangzhou, Zhejiang, P.R. China; ^4^ Department of Gastrointestinal Surgery, Shaoxing People's Hospital, Shaoxing Hospital of Zhejiang University, Shaoxing, Zhejiang, P.R. China

**Keywords:** gut microorganism, microbiota-gut-brain axis, central nervous system, disorders

## Abstract

The gut and brain form the gut-brain axis through bidirectional nervous, endocrine, and immune communications. Changes in one of the organs will affect the other organs. Disorders in the composition and quantity of gut microorganisms can affect both the enteric nervous system and the central nervous system (CNS), thereby indicating the existence of a microbiota-gut-brain axis. Due to the intricate interactions between the gut and the brain, gut symbiotic microorganisms are closely associated with various CNS diseases, such as Parkinson's disease, Alzheimer's disease, schizophrenia, and multiple sclerosis. In this paper, we will review the latest advances of studies on the correlation between gut microorganisms and CNS functions & diseases.

## INTRODUCTION

### Gut microorganisms

The human gut contains various microorganisms, such as bacteria, fungi, parasites, and viruses, and more than 100 million bacteria reside in human gastrointestinal tract, which is 10-100 times the number of eukaryotic cells in our body. After years of common development with the human body, the gut bacteria have reached a mutually beneficial symbiotic state with the human body. The gut bacteria mainly include six major phyla of Firmicutes, Bacteroidetes, Proteobacteria, Actinomycetes, Verrucomicrobia, and Fusobacteria, with Bacteroidetes and Firmicutes being the dominant flora [1].

Gut microorganisms play an important role in promoting adult development and homeostasis; for example, they can affect human metabolic functions by decomposing the complex polysaccharides in food. In addition, gut microorganisms can regulate gut movement, the gut barrier system and fat distribution [2]. Gut microorganisms can affect immune function through the development of gut-associated lymphoid tissue and by preventing the colonization of pathogens, and they can affect the energy metabolism and mitochondrial function of the host. The intricate relationship governing host and microorganism interactions suggest that when this relationship is abnormal, the microorganisms may cause the pathogenesis of disease or promote the progression of disease. Therefore, recent research has focused on determining the diversity of these microorganisms to clarify the physiological roles they play and eventually to prevent and treat diseases by controlling the microorganism species. For example, recent studies demonstrated that gut bacteria can enhance the efficacy of commonly used chemotherapy drugs [3].

Age and diet, in particular, can have a significant impact on gut microbiota [4, 5], and numerous human and animal studies have demonstrated that different diets can cause a significant change in the microbiota. For example, the species of colonized bacteria in the gut of Europeans significantly differs from that of people from rural Africa due to the difference in their daily diets [6]. By examining the stool samples of 98 people, Wu et al. [7] found that Bacteroides were mainly associated with a diet that was high in animal fat and proteins, whereas Prevotella was mainly associated with a high-carbohydrate diet. Infection and disease can also have an adverse effect on the normal gut flora of the host, thereby causing deleterious effects on the host; for example, disrupting gut microorganisms in rats will cause long-term visceral pain and stress-related disorders, such as irritable bowel syndrome [8]. The microbiota organisms, which are symbiotic with the human body, constitute a complex micro-ecological system, and a change in the quantity and quality of the gut microorganism population may affect the gut barrier function, increase the secretion of toxic substances, and decrease the secretion of substances that are beneficial to human body, leading to enteral and external diseases.

There are three main methods for detecting gut microorganisms: the bacteria culture technique, the traditional molecular biology technique that is independent of culture, and high-throughput sequencing technology. The former is mainly used for stool culture, this method is time-consuming, and the bacterial species obtained are limited. The latter two mainly isolate the bacterial DNA from the stool for the detection, the detection is fast, and the bacterial species are complete.

### Microbiota gut-brain axis

The central nervous system (CNS) is closely related to the gastrointestinal tract, and the CNS plays an important role in regulating gut function and homeostasis. In turn, the gut flora may affect the CNS and nerve cells, participate in the regulation of nervous system function, affect the pathogenesis and progression of nervous system-related diseases. Due to the complex relationship between the gut microorganism population and the host, the authors proposed a new concept: the microbiota gut-brain axis. The microbiota gut-brain axis is the focus of recent research on the gut microecology. In addition to studies of the relationship between the gut microecology and neurological function, recent studies have emphasized how this relationship affects human health.

The brain and gut can be connected through a variety of pathways, including the enteric nervous system (ENS), vagus nerve, the immune system, or the metabolic processes of gut microorganisms.

The gastrointestinal tract is an organ that is co-dominated by the CNS, the autonomic nervous system, and the ENS. Regulation of enteric nerves consists of four levels of nervous regulation [9]. The first level is the local regulation by the ENS. The ENS consists of two nerve plexuses, the myenteric and submucosal plexuses, and the motor neurons and the sensory neurons of the ENS connect to each other to perform an independent information integration and processing function, which is similar to the situation for the brain and the spinal cord. The second level is located at the prevertebral ganglia, which receives information transmitted from both ENS and CNS nerves. The third level is the CNS. After integrating various pieces of information from various centers of the brain and spinal cord when they receive signals regarding internal or external environmental changes, the CNS transmits its regulatory information to the ENS or directly acts on gastrointestinal effector cells through the autonomic nervous system and the neuroendocrine system to regulate the smooth muscle, glands and blood vessels. The fourth level consists of advanced brain centers; information from the cortex and the subcortical region converges downward to specific brain stem nuclei from the basal ganglia. This type of neuroendocrine network, which connects the gastrointestinal tract with the CNS at different levels, is the structural basis for the function of the microbiota gut-brain axis. Disorders of neurological control at any level will affect the function of the gut and brain. The gut has a direct neural connection with the brain through the vagus nerve, and bacteria can stimulate the afferent neurons of the ENS [10]. Disorders of the microbiota gut-brain axis are associated with depression, anxiety, irritable bowel syndrome, inflammatory bowel disease, CNS diseases and other diseases.

The vagus nerve of the body can control the function of multiple organs, such as heart rate and gut motility; the vagus nerve can also transmit peripheral immune signals to the CNS. The vagus signal from the gut can trigger an anti-inflammatory response against the sepsis induced by microorganisms. Gut microorganisms can affect brain functions through the vagus nerve; after a vagotomy, the microorganisms will not be able to regulate behaviors [11]. After vagotomy in mice, no behavioral change was found even for mice that were treated with probiotics. Similarly, the bifidobacteria treatment, which had been previously reported to be effective, did not improve the behavior of vagotomized mice [12].

Because gut microorganisms can directly affect the immune system, immune activation may be the pathway for transmitting microbial actions to the CNS [13]. Microorganisms can also enhance the anti-tumor immune effect of drugs by promoting T cell accumulation and transformation [14, 15], and microorganisms are very important for the immune function of organisms. The immune system plays an important role in maintaining health by maintaining gut homeostasis. The elderly have a decreased immune function, resulting in a change in the microorganism-brain connection and subsequent behavioral change. Microglia are immune cells in the CNS, and studies have found that the metabolism of gut microorganisms can regulate the maturation and function of microglia, thereby affecting CNS function [16].

Microorganisms can also cause neurophysiological changes in the host by producing chemical substances that bind to the receptors inside and outside of the gut. Bravo et al. found that mice fed L. rhamnosus (JB-1) probiotics exhibited fewer anxiety-like and depressive behaviors and a change in the GABA Aα2 mRNA content in the brain area that controls specific behaviors [11]. Short-chain fatty acids (SCFAs) are produced from the dietary fibers fermented by gut microorganisms in the large intestine. In animal models, SCFAs can improve the neurodevelopment and cognitive function of animals with neurodegenerative disorders [17]. However, Macfabe injected a specific type of SCFA, i.e., propionic acid (PPA), into rats, which then exhibited autism-related characteristics and neurochemical changes [18]. These neurochemical changes included neuroinflammation, increased oxidative stress, and depletion of antioxidants, thereby leading to mitochondrial dysfunction. Other microorganism-derived molecules, such as the neural activation molecules serotonin, melatonin, histamine, and acetylcholine also have a role in the microbiota gut-brain axis [19].

In addition, studies have shown that the microbiota may affect the CNS by altering adult hippocampal neurogenesis (AHN). The adult hippocampus and lateral ventricle have the function of generating new neurons. AHN has a role in learning and memory and can have affect on the pathogenesis of many neurological disorder-related diseases and symptoms, such as epilepsy, depression, Alzheimer's disease (AD), and Parkinson's disease (PD) [20]. In a recent study, Ogbonnaya et al. found a difference between the hippocampal neurons of sterile mice and those of normal mice and that microbiota colonization after weaning did not alter the amount of AHN, suggesting that early in life, microorganisms play an important role in AHN [21].

### Microorganisms and brain function

The impact of microorganisms on behavior and cognition has been increasingly recognized. Microbial signals can regulate important functions of healthy human bodies, and growing evidence has demonstrated that many diseases are due to disturbances of gut microorganisms [22]. Early studies in animals showed that the introduction of single, unique flora could lead to the development of anxiety-like behavior, and this change was accompanied by the activation of neurons in the brain that relied on the gut information transmitted to the brain via the vagus nerve [23]. A subsequent study found that colonization of the fecal microorganisms from the mice of a certain behavior type into another type of mice could cause the receptor mice to show behavior similar to that of the donor mice. When the microorganisms of specific-pathogen-free (SPF) Swiss mice were transferred to the bodies of sterile BALB/c mice, the exploration behavior of sterile BALB/c mice increased; however, when the microorganisms of SPF BALB/c mice were transferred to the body of sterile Swiss mice, the exploration behavior of sterile Swiss mice became lower than that of normal Swiss mice, thus indicating that behavior type may be directly associated with microorganisms [24].

In 2004, Sudo et al. were the first to report that symbiotic gut microorganisms were associated with the hypothalamic-pituitary-adrenal (HPA) axis [25]. In response to stress, the levels of corticosteroid hormone and adrenal hormones were higher in sterile mice than in mice harboring normal microorganisms. Gut colonization by Bifidobacterium can attenuate the increased HPA response; however, this inhibition can only be initiated in the early stages of life, indicating that the most primitive exposure to microorganisms is necessary for the inhibition of neural regulation by the HPA axis. The HPA axis is very important for learning and memory, and an HPA disturbance can lead to impairment of hippocampal memory. The brain-derived neurotrophic factor (BDNF), N-methyl-D-aspartate (NMDA), c-fos levels are reduced in sterile mice [26], and these substances have a role in memory retention, cognitive function, and other brain functions.

The change in gut microorganisms found in sterile animals or with the use of probiotics, antibiotics, and colonization with fecal microorganisms can influence the cognitive function of the host. For example, supplementation with probiotics for a week prior to infection can not only prevent the microorganism disturbance caused by the infection but also prevent the changes in cognitive behavior caused by stress [26]. Liang et al. found that probiotics could significantly improve the cognitive dysfunction induced by chronic restraint stress [27]. In a human experiment, fMRI tests found that the activities in the brain regions that control brain memory and the processing of sensation were altered after female volunteers consumed fermented milk that contained probiotics [28]. The above various studies found that the stability of the equilibrium state of normal microorganisms in the gut was closely related to brain development and function.

### Microbiota-gut-brain axis and CNS disorders

#### Microbiota-gut-brain axis and neuropsychological disorders

Schizophrenia is a neuropsychological disorder, and whole-genome analysis suggests that immunity-related genes may be changed in schizophrenia patients. Song et al. [29] found that increases in the immune response and inflammatory state in schizophrenia patients. Microorganisms can regulate BDNF and NMDA receptors [25], and these neurotrophic factors and proteins have a role in brain development and neural plasticity. The change in BDNF expression leads to the cognitive dysfunction of schizophrenia patients, and antagonists of the NMDA receptor can produce schizophrenia symptoms. When the NMDA receptor function in a host is enhanced, the patients’ symptoms can be relieved, and their cognitive ability can be improved [30]. Microorganisms and intestinal mucosal cells can regulate the changes in chemical factors, such as the proinflammatory interleukin-8 (IL-8) and IL-1 or the anti-inflammatory IL-10 and transforming growth factor β (TGFβ). The serum levels of proinflammatory cytokines in schizophrenia patients are higher than those of normal controls, and the levels of serum inflammatory markers are positively correlated with the clinical symptoms of schizophrenia patients [31]. Additionally, the ratio of T helper cell type 1 (Th1) to Th2 in schizophrenia patients is increased, suggesting that the inflammatory response in the patients is increased; after an effective treatment with drugs, this ratio will be corrected [32]. After female rats were fed Zyprexa, a significant change in gut bacteria was detected, including an increase in Firmicutes and a decrease in Bacteroidetes [33], and the levels of systemic inflammatory response markers in these rats, such as IL-6, IL- 8, tumor necrosis factor-α (TNF-α) and IL-1β, increased significantly.

Gut bacteria can also produce harmful substances that damage the intestinal epithelial barrier, causing neurotoxic bacterial products and proteins to enter the circulatory system [34]. Severance et al. [35] found that the concentration of antibodies against saccharomyces cerevisiae was higher in the body of schizophrenia patients, and this genus was a marker of gut inflammation. An increase in the levels of circulating pathogen antigen can cause the host to respond to its own tissues and cells through a form of molecular induction [36], and this response is the central process of autoimmune diseases. Compared with the normal population, schizophrenia patients have a higher probability of developing autoimmune disorders, and specific brain regions of schizophrenia patients, such as the hippocampus, amygdala, and frontal cortex, have higher levels of autoimmune antibodies [37]. Gut symbiotic flora can cause an immune response that produces more Th17 cells, leading to the occurrence of an autoimmune response. Additionally, schizophrenia patients have a higher proportion of Th17 cells [38]. Krych L et al. [39] found that rodents that received a long-term treatment with phencyclidine would show schizophrenia-like behavior, and this behavior was accompanied by a change in the core gut microorganisms. When ampicillin was used to reduce the gut microorganisms, the cognitive behavior of the animals could be corrected. The above experiments show the impact of changes in gut microorganisms on schizophrenia.

Autism spectrum disorder (ASD) is a type of extensive brain function development disorder that begins during the early stages of growth (< 36 months), and it is manifested as different levels of interpersonal barriers, language development disorders, and stereotyped behavior. Environmental factors (especially gut dysbacteriosis) are involved in the development of ASD, and as many as 70% of autistic patients exhibit gastrointestinal tract-related symptoms; therefore, it is thought that ASD is associated with impairment of the gut-brain axis [40]. The majority of autistic patients have diarrhea, abdominal pain, constipation, gastroesophageal reflux, and other gastrointestinal symptoms. Mazurek et al. studied 2,973 ASD patients and found that 24% of the patients had at least one type of gastrointestinal disease [41], and Adams et al. found that ASD child patients showed significantly greater occurrence of diarrhea, abdominal distension, and constipation than normal children, and the gastrointestinal symptoms were also closely associated with the severity of ASD [42]. Moreover, ASD patients have a significantly higher content of the genera Faecalibacterium and Clostridium in the gut than normal children [43, 44]. Desbonnet et al. placed sterile mice and normal mice in two separate spaces, and the sterile mice would develop autistic behavior, whereas recolonization of symbiotic flora was able to restore the partially defective social behavior in the mice [45]. Impaired immune function is a common feature of ASD patients, and the increase in the genera Faecalibacterium may be the main reason for immune dysfunction in ASD patients [43].

Obese pregnant mice exhibit changes in social behavior, oxytocin cells, synaptic plasticity, and the microorganism composition of the microbiota, but Lactobacillus reuteri could reverse these changes [46]. Because gut flora imbalance can increase the permeability of the intestinal mucosa, the metabolic products of gut microorganisms and certain cytokines can enter the circulation system and cause damage to the CNS, resulting in a delay in the early neurological development in child patients. Christensen used metabolomics to identify a large number of bacteria-derived metabolites that might be associated with ASD-related behaviors [47]. Adams found that the levels of SCFAs, which are very important for the development of neurological functions, in the stool samples of autistic children were lower than those of normal children [42]; similarly, the PPA levels were also reduced. The exposure of pregnant rats to PPA before giving birth impaired the social behavior of their newborn and adolescent rats [48]. Moreover, following PPA injection into the brain ventricles of adult rats, the rats exhibited a behavioral change similar to that of human ASD, and the injection also induced changes in ASD-related genes, including genes for neurotransmitters, nerve cell adhesion factors, and oxidative stress [18].

The bacterial metabolites, 4-ethyl-phenylsulfate and 3-(3-hydroxy phenyl)-3-hydroxypropionic acid, can cause mice to develop ASD-like symptoms [49, 50]. In addition, the serum lipopolysaccharide (LPS, cell wall components of gram-negative bacteria) levels are significantly higher in ASD patients than in normal subjects, and LPS has a clear association with social disorders [51]. Most children with ASD have a history of infection before age 2, and the frequency of their use of antibiotics is also significantly higher than that of normally developed children [52]. Antibiotics destroy the physiological balance of the inherent gut flora, and the newly colonized microorganisms produce neurotoxins, thereby inducing chronic diarrhea and ASD. For example, an abnormal increase in Clostridia and Bacteroidetes in the gut can promote gastrointestinal symptoms and ASD behaviors [18]. Therefore, both the imbalance of gut flora and the entry of excessive amounts of bacterial metabolites into the brain through the circulation are associated with the onset of ASD.

### Microbiota-gut-brain axis and neurodegenerative diseases

Multiple sclerosis (MS) is a chronic inflammatory demyelinating disease of the nervous system, and its etiology is still unclear. MS is associated with a significant increase in the number of cells that have immune response to the patients’ own nervous system because gut microorganisms play an important role in the development of the autoimmune system and are associated with a variety of autoimmune and metabolic diseases. Therefore, it is speculated that gut symbiotic microorganisms play an important role in the susceptibility to MS.

Experiments have been performed to study the role of gut microorganisms in an animal model MS-experimental allergic encephalomyelitis (EAE). Ochoa-Reparaz et al. [53] used a mixture of antibiotics to alter gut microorganisms before inducing EAE in mice, and they found that treatment with orally administered antibiotics could significantly reduce fecal and gut microorganisms and could markedly impair the development of EAE and mitigate the severity of EAE, compared with those for the intraperitoneal injection treatment and no treatment. Other studies showed that when the mice were treated with orally administered antibiotics, the production of the anti-inflammatory factor IL-10 was increased, thereby improving EAE. Sterile mice may be immune to EAE, and even when they do have EAE, their disease course is milder [54]. Sterile mice have a relatively lower number of interferon-γ (IFN-γ)-producing CD4+ Th1 cells and IL-17A-producing CD4+ Th17 cells in their body, and these two substances are both very important for the development of EAE. Re-colonization of segmented filamentous bacteria into sterile mice can significantly increase the severity of EAE and increase the number of Th1 and Th17 cells in the nervous system. When spontaneous EAE transgenic mice were housed in a sterile environment, these mice did have EAE, and the number of Th17 cells in the gut lymph node tissues decreased. Moreover, the amount of IL-17 and IFN-γ produced by the cells also decreased.

The drug resistance of ε-toxin in the plasma of MS patients is also higher than that of normal people. Vartanian et al. [55] found the colonization of Clostridium perfringens type B in patients with recurrent MS, and the ε-toxin produced by this pathogen could cause damage to the blood-brain barrier (BBB), thereby damaging neurons and oligodendrocytes [56]. Consequently, Clostridium perfringens type B is thought to be the initiating factor for susceptible individuals to experience the pathological change of autoimmune demyelination in the future. In addition to the toxic effects, MS patients may have an imbalance of various bacteria of the Bacillus genus and species. For example, Jhangi et al. [57] found that MS patients had an increase in the genus Methanobrevibacter, whereas the Lachnospiraceae content was reduced. Tremlett H et al. [58] found that child MS patients had an increase in Desulfovibrio, whereas the Lachnospiraceae and rumen bacteria contents were decreased. These results suggest that gut microorganisms can affect the parenteral and CNS immune response and self-flora imbalance to promote the development and progression of EAE.

PD is an extensive neurodegenerative disease, its pathogenic mechanism is complex, and a change in α-synuclein is an important pathogenic mechanism of this disease. Changes in the gut microbial composition may affect the gut barrier function and gut permeability, thereby affecting intestinal epithelial cells and the immune system. The primed immune response initiated by gut microorganisms can enhance the inflammatory response to brain amyloid (α-synuclein), thereby causing the misfolding of α-synuclein [59]. Fireland [60] reported that bacterial proteins could cause inflammation, oxidative stress, and cytotoxicity and consequently induce or affect the progression of PD. The neuroinflammatory response in PD patients is associated with the upregulation of Toll-like receptor-2 (TLR2) and the activation of microglia [61]. In animal models of PD, the peripheral inflammatory response can induce the complement pathway in microglia and cause damage to dopaminergic neurons [62].

Proinflammatory factors associated with chronic bowel diseases can induce intracranial inflammation, lead to the death of dopaminergic neurons, and eventually cause the development of PD [63]. Scheperjans F et al. [64] found that PD patients had a decrease in Prevotella. By comparing the stools of 34 PD patients and 34 normal controls, Unger MM et al. [65] found that PD patients had a decrease in Bacteroidetes and Prevotella in their stool, which was accompanied by a reduction in SCFAs. As symbiotic gut bacteria, Prevotella are involved in the mucus formation of the mucosal layer of the gut and the production of the neuroactive SCFAs through fiber fermentation. The reduction of Prevotella causes a decrease in gut mucus and an increase in gut permeability, increasing local and systemic susceptibility to the influence of bacterial antigens and endotoxins, thereby inducing the expression and misfolding of a large amount of *α-synuclein. The* inflammatory changes observed in PD patients and PD animal models are associated with increased gut permeability [66]. LPS is a gut-derived proinflammatory bacterial endotoxin that can cause changes in the substantia nigra, and it can act as a PD-promoting substance [67]. Similarly, Keshavarzian A et al. [68] used high-throughput sequencing technology to examine the stool samples from 38 PD patients and 34 healthy individuals, and they found that the LPS synthesis gene was significantly higher in PD patients than in normal controls.

AD is a degenerative disease of the CNS, its onset is recessive, and its disease course is chronically progressive. The pathological markers of AD include extracellular β-amyloid (Aβ) senile plaques and intracellular neurofibrillary tangles. The number and maturation of microglial cells in sterile mice are abnormal, resulting in damage to the immune system and ultimately leading to the development of neurological diseases, such as AD [16]. Cognitive behavior impairment is a characteristic of AD patients, and the influence of gut microorganisms on cognitive behavioral capability has demonstrated the role of gut microorganisms in the pathogenesis of AD. Bruce-Keller fed C57BL/7 mice with a normal diet and a high-fat diet, and the mice in the high-fat diet group would show impairment in cognitive behavior. When the gut microorganisms in the high-fat diet mice were transferred to the mice who had a normal body weight but had an antibiotics-induced paucity of microorganisms, the mice that received the gut microorganisms from the high-fat diet group mice showed selective impairment in exploratory behavior, cognitive behavior, and gut permeability, and their systemic and central inflammatory responses increased [69]. This finding suggests that the behavior changes induced by a high-fat diet may be due to changes in gut microorganisms, and the microorganisms in turn change the cognitive behavioral capacity.

The integrity of the BBB is important for brain function and development. The inflammation caused by the changes in gut microorganisms will lead to changes in BBB integrity, which in turn affects brain function. Under normal conditions, LPS cannot enter the bloodstream due to the tight junction between intestinal epithelial cells. However, when the tight junction of cells is disrupted and the permeability is increased, LPS can enter the bloodstream and induce inflammatory response. Studies found that the plasma LPS concentration in AD patients is three times that of normal patients [70]. Furthermore, intraperitoneal injection of LPS into mice can cause an Aβ-protein increase in hippocampus, cognitive defects, and memory impairment [71]. The increase of the inflow and the decrease in the outflow of the Aβ protein in AD patients cause the aggregation of the Aβ protein in AD patients, and this finding suggests a decrease in the capacity to clear the Aβ protein and an increase in BBB permeability in AD patients [72]. The increased concentration of plasma LPS in AD patients implies an impairment of the gut barrier function and increased gut inflammation and permeability, which further suggests that gut microbiota may participate in the pathophysiological process of AD.

Gut microorganisms can also affect brain functions through the synthesis of various substances. Serotonin is very important for cognitive function, 95% of serotonin is synthesized in the gut, and gut microorganisms play an important role in serotonin synthesis. The gut serotonin level in sterile mice was 60% lower than the normal value [73]. The use of serotonin reuptake inhibitors can reduce Aβ-protein levels in the brain, indicating that serotonin can reduce the formation of Aβ plaques, thereby reducing the risk of AD [74]. In sterile mice, BDNF was significantly reduced, and this change was accompanied by cognitive function changes. Similarly, in AD patients, the BDNF levels in the brain and in the serum were significantly reduced [75]. The Aβ production and clearance in the CNS is a dynamic change, and some bacteria and fungi can secrete amyloid, resulting in an increase of amyloid levels in the CNS that disrupts the dynamic balance of the Aβ protein, which leads to Aβ-protein aggregation in the brain and a high AD risk [76]. Therefore, an imbalance in gut microbiota may promote the development of AD by affecting intestinal function and the synthesis and secretion of substances.

### Summary

The interactive relationship between the brain and the gut includes neurology, metabolism, hormones, immunity, and other aspects, and changes in any component may lead to a functional change in the two interactive systems. The normal ecological balance of gut microorganisms plays an important role in the maintenance of this relationship. Microorganisms affect the development and function of the CNS through the microbiota-gut-brain axis. The mechanisms of many CNS diseases are still unclear, and the discovery of this complex relationship, the microbiota gut-brain axis, has provided a new research direction for the study of CNS diseases that do not have a clear pathogenic mechanism.
